# An Emerging Role of micro-RNA in the Effect of the Endocrine Disruptors

**DOI:** 10.3389/fnins.2016.00318

**Published:** 2016-06-30

**Authors:** Adel Derghal, Mehdi Djelloul, Jérôme Trouslard, Lourdes Mounien

**Affiliations:** ^1^Aix Marseille University, PPSNMarseille, France; ^2^Department of Cell and Molecular Biology, Karolinska InstituteStockholm, Sweden

**Keywords:** micro-RNA, endocrine disruptors, environment

## Abstract

Endocrine-disrupting chemicals (EDCs) are diverse natural and synthetic chemicals that may alter various mechanisms of the endocrine system and produce adverse developmental, reproductive, metabolic, and neurological effects in both humans and wildlife. Research on EDCs has revealed that they use a variety of both nuclear receptor-mediated and non-receptor-mediated mechanisms to modulate different components of the endocrine system. The molecular mechanisms underlying the effects of EDCs are still under investigation. Interestingly, some of the effects of EDCs have been observed to pass on to subsequent unexposed generations, which can be explained by the gametic transmission of deregulated epigenetic marks. Epigenetics is the study of heritable changes in gene expression that occur without a change in the DNA sequence. Epigenetic mechanisms, including histone modifications, DNA methylation, and specific micro-RNAs (miRNAs) expression, have been proposed to mediate transgenerational transmission and can be triggered by environmental factors. MiRNAs are short non-coding RNA molecules that post-transcriptionally repress the expression of genes by binding to 3′-untranslated regions of the target mRNAs. Given that there is mounting evidence that miRNAs are regulated by hormones, then clearly it is important to investigate the potential for environmental EDCs to deregulate miRNA expression and action.

## Introduction

Endocrine-disrupting chemicals (EDCs) are diverse natural and synthetic chemicals that may alter various mechanisms of the endocrine system and produce adverse developmental, reproductive, metabolic, and neurological effects in both humans and wildlife (Henley and Korach, 2006). To date, close to 800 chemicals are known or suspected to be capable of interfering with hormone receptors and/or hormone synthesis and then play a larger role in the causation of many endocrine diseases and disorders (WHO | State of the science of endocrine disrupting chemicals, 2012). Excretion of EDCs is dependent on the nature of the chemical substances. If the substance is non-persistent it is usually predicted that they are metabolized by the liver then finally eliminated from the body through feces and urine. Persistent endocrine disruptors are accumulated especially in adipose tissue and they can be released slowly. One way of excretion of these persistent endocrine disruptors is thought to be from mother to child through breast feeding. It is observed in many studies that the daily intake of breast milk containing organic pollutants may exceed the tolerable limit. It has been established that some EDCs can act directly on hormone receptors as hormone mimics or antagonists. Others can act directly on proteins that control the delivery of a hormone to its target cell or tissue. In addition, EDCs may act synergistically and produce additive effects. Most studies on EDCs have focused on chemicals that affect the reproductive and thyroid axis. However, several studies have suggested that environmental chemicals could affect several physiological systems that lead to metabolic disorders or central nervous system dysfunctions (Casals-Casas and Desvergne, 2011). For instance, neurobehavioral disorders have been associated with hypothalamic-pituitary-adrenal (HPA) axis disruption induced by hydroxyl-polychlorinated biphenyl (PCB; Kimura-Kuroda et al., 2007).

It is particularly difficult to highlight only one mechanism of action shared by the set of EDCs. In fact, the main problem is that there are many and diverse EDCs including industrial chemicals, pesticides, pollutants, and plastic industry compounds. Nevertheless, research on EDCs has revealed that they use a variety of both nuclear receptor- and non-receptor- mediated mechanisms to modulate different components of the endocrine system. For instance Vinclozolin (VCZ), a widely used fungicide with antiandrogenic effects in mammals, is a competitive antagonist of androgen receptor (AR) ligand binding (Kelce et al., 1997). Several studies showed that exposure to VCZ induce masculinized females and feminized males in rodents (Buckley et al., 2006). Interestingly, some of the effects of VCZ have been observed to pass on to subsequent unexposed generations, which can be explained by the gametic transmission of deregulated epigenetic marks (Anway et al., 2005; Stouder and Paoloni-Giacobino, 2010; Guerrero-Bosagna et al., 2012; Skinner et al., 2013). Epigenetic mechanisms, including histone modifications, DNA methylation, and specific micro-RNAs (miRNAs) expression, have been proposed to mediate such transgenerational transmission (Reik et al., 2001; Del-Mazo et al., 2013).

This review provides an insight into the toxicological effects of EDCs and particularly new molecular mechanisms, i.e., miRNAs, involved in the EDCs induced endocrine disruption.

## The different types of EDCs

The term endocrine disruptors were first introduced by the group of Soto in 1993 that showed that EDCs induced developmental abnormalities (Colborn et al., 1993). The International Program on Chemical Safety (IPCS) in 2002 and World Health Organization in 2013 defined EDCs as “…*an exogenous substance or mixture that alters function(s) of the endocrine system and consequently causes adverse health effects in an intact organism, or its progeny, or (sub) populations. A potential endocrine disruptor is an exogenous substance or mixture that possesses properties that might be expected to lead to endocrine disruption in an intact organism, or its progeny, or (sub) populations*.” To date, EDCs include a large variety of chemical classes such as pesticides [methoxychlor, chlopyrifos, and dichlorodiphenyltrichloroethane (DDT)], pharmaceutical agents [diethylstrilbestrol (DES)], plastic packaging compounds [Bisphenol A (BPA), phthalates], and other industrial products that are used in daily life as fungicides VCZ or solvents/lubricants (dioxins). Some of them but not all are exposed in this paragraph.

A large number of chemicals are used as pesticides. The most important pesticides are organochlorines pesticides (OCPs), organophosphates, or triazines. The most emblematic of the banned OCPs is DDT and the exposure to it persists. The pesticides are involved in a large number of diseases including cancer, diabetes but also neurodegenerative disease as Parkinson or Alzheimer (Mostafalou et al., 2012; Mostafalou and Abdollahi, 2013).

The dioxins are a general name for a family of organochlorines including the polychlorinated dibenzodioxins (PCDDs), the polychlorinated dibenzofurans (PCDFs), and the polychlorinated biphenyls (PCBs). Dioxins are produced by various industrial processes and are commonly regarded as highly toxic compounds that are environmental pollutants and persistent organic pollutants. Among the PCDDs, the 2,3,7,8-Tetrachlorodibenzo*-p-*dioxin (TCDD) is the most potent and toxic compound and became known as a contaminant in Agent Orange, a herbicide used as a weapon in the Vietnam War (Schecter et al., 2006). TCDD was also released into the environment during the Seveso disaster (Sweeney and Mocarelli, 2000). The TCDD and the other toxins have been shown to be involved in different diseases including cancers, thyroid dysfunction, and nervous system degeneration but also type 2 diabetes (Pelclová et al., 2006; Schecter et al., 2006; Mostafalou et al., 2012; Mostafalou and Abdollahi, 2013).

An important number of EDCs are found in plastic products. World plastic production exceeded 300 million tons in 2010 (Halden, 2010). Most abundant of these plastics are phthalates and BPA. These are two most common EDCs and are associated with parental and social behavioral disturbances but also endocrine disease. Phthalates are mainly used as plasticizers in a wide range of common products, and are released into the environment. Phthalate exposure may be through direct use or by indirect means through leaching and general environmental contamination (Aurela et al., 1999). Food products are believed to be the main source of di-(2-ethylhexyl) phthalate (DEHP) and other phthalates in the general population. Fatty foods such as milk, butter, and meats are a major source. In several studies in human and rodents, high and even low doses of phthalates have been shown to change hormone levels as T3, T4, and thyroid-stimulating hormone and cause birth defects (Gayathri et al., 2004; Heudorf et al., 2007; Meeker et al., 2009). BPA is one of the other emblematic plastics used in polycarbonate plastic and polystyrene resins. Interestingly, it has been shown that BPA is detected in 95% of urine sample from a reference population of 394 adults in the United States (Calafat et al., 2005). This higher level of BPA in urine is associated with cardiovascular disease, sterility, and other reproductive diseases but also diabetes and liver abnormalities (Takeuchi et al., 2004; Sugiura-Ogasawara et al., 2005; Lang et al., 2008).

## The toxicological effects of EDCs on endocrine axis

### Reproductive axis

In the last few years, it has been noticed that the incidence of certain diseases of the reproductive axis has increased (WHO | State of the science of endocrine disrupting chemicals, 2012). It is well-established that estrogen and androgen are involved in sexual differentiation. In this context, EDCs may act as estrogen and or androgen antagonists and induce different sexual disorders in males and females (Diamanti-Kandarakis et al., 2009; Sweeney et al., 2015; Toppari et al., 2016). For instance, DES and phthalates exposure to rats are associated with cryptorchidism or micropenis (Fisher et al., 2003; Li et al., 2003). In human, it has been shown that breast milk dioxin concentration correlated positively with the risk of cryptorchidism in Denmark (Main et al., 2007). It has also been shown that perinatal exposure to low doses of dioxin can permanently reduce sperm quality (Mocarelli et al., 2011). In humans, exposure to PCBs caused a defect in the development of the reproductive system (Staessen et al., 2001). Recently, epidemiological, study suggested that prenatal exposure to PCBs may be also associated with increased risk for cryptorchidism (Koskenniemi et al., 2015).

EDCs are associated with some types of female reproductive axis disorder including polycystic ovarian syndrome (PCOS). PCOS is a problem in which a woman's hormones are out of balance. It can disrupt the menstrual cycle and makes it difficult to become pregnant. If it isn't treated, over time it can lead to serious health problems, such as diabetes and heart disease. Most women with PCOS grow many small cysts on their ovaries. Interestingly, women with PCOS have higher levels of BPA and increased testosterone in these women is consistent with decreased clearance of BPA (Takeuchi et al., 2004, 2006). The cause of PCOS is not fully understood, but the EDCs as well as BPA could play a role in the onset of PCOS. Female rats exhibited sexual precocity as a consequence of exposure to DTT (Rasier et al., 2007).

It has also been shown in the hypothalamic GT1-7 cell line that organochlorine pesticides such as methoxychlor and chlopyrifos altered gonadotropin-releasing hormone (GnRH) gene expression and biosynthesis (Gore, 2002) suggesting that EDCs could affect the different levels or reproductive axis. Interestingly, it has been revealed that the BPA-mediated inhibition of GnRH neuronal activity occurred independent of estrogen receptors via a non-canonical unknown pathway (Klenke et al., 2016).

### Thyreotropic axis

Thyroid hormones (T3 and T4) are important for brain development, for the modulation of metabolism and are associated with many aspects of normal adult physiology. For these reasons, thyreotropic axis disruption induced a large scale of perturbation in adult physiology, development, and metabolism. It has been reported that numerous EDCs can directly affect the normal functioning of the thyroid gland. In numerous studies, it has been shown that different EDCs such as PCBs, BPA, or DTT have thyroid-disrupting effects in animals and humans (Patrick, 2009; Molehin et al., 2016).

The EDCs can affect the thyroid system at different levels such as the transport and/or biosynthesis of the thyroid hormones. It has been shown that PCBs have a high affinity with thyroxin specific binding protein which can affect the thyroid hormone transport (Rickenbacher et al., 1986; McKinney et al., 1987; Darnerud et al., 1996). More precisely, treatment of mice during gestation with PCB as 3,3′, 4,4′-tetrachlorobiphenyl (CB-77) leads to a decrease of free and total T4 in fetal plasma (Darnerud et al., 1996). More recently, the group of Seegal examined the effects of a mixture of PCBs and polybrominated diphenyl ethers (PBDEs) coexposure from gestational day 6 through postnatal day 21, alone and in combination, on T4 levels in rat offspring (Miller et al., 2012, 201). They observed that PCBs and PBDEs induces similar reductions in T4 levels and that coexposure to a mixture of PCBs and PBDEs has additive effects on T4 level in male and female offspring (Miller et al., 2012). In the study of Schmutzler et al., rats (female, ovariectomized) were treated for 12 weeks with different EDCs and an alteration in thyrotropin (TSH) and thyroid hormones (T4, T3) serum levels were observed (Schmutzler et al., 2004). In another set of studies, exposure to phthalates induced thyroid function alterations (Mitchell et al., 1985; Hinton et al., 1986; Price et al., 1988). Interestingly, the treatment of rats for periods of 3 months with di-(2-ethylhexyl) phthalate increased the number and size of lysosomes, hypertrophy of the Golgi apparatus, and dilation of the rough endoplasmic reticulum in thyroid cells and these changes are consistent with persistent hyperactivity in the gland (Price et al., 1988). It has also been shown that EDCs can alter deiodinase activity which is the peroxidase enzyme that is involved in the activation or deactivation of thyroid hormones (Meerts et al., 2002; Viluksela et al., 2004; Noyes et al., 2013).

In human, there is now growing evidence that PCBs but also BPA and phthalates have thyroid-disrupting effects (Boas et al., 2012; Campos and Freire, 2016). For instance, the group of Yoshinaga showed that exposure to hydroxylated-PCBs at environmental levels during the first trimester of pregnancy can affect neonatal thyroid hormone status (Hisada et al., 2014). It has also been shown that early exposure to certain environmental chemicals with endocrine-disruption activity as pesticides may interfere with neonatal thyroid hormone status (Freire et al., 2011).

### Central nervous system

There is strong evidence that there is a correlation between the increasing prevalence of neurodevelopmental disorders and the increase in exposure to pollutants over the past several decades (Weiss and Landrigan, 2000; Landrigan and Goldman, 2011a,b). For instance, since the 1970s, there have been dramatic increases in previously rare neurodevelopmental disorders such as autism which is characterized by some degree of impaired social behavior, communication and language, and a narrow range of interests and activities that are both unique to the individual and carried out repetitively. In the 1970s, autism's prevalence was estimated to be between 4 and 5 in 10,000 children (Wing et al., 1976) but today this value is estimated to be 1 in 110 children (Rice et al., 2007). In a review of the literature performed by de Cock et al., a positive association was found for autism in relation to exposure to different chemicals investigated, which included hazardous air pollutants, pesticides, and BPA (de Cock et al., 2012). In the same study, a relationship between attention deficit hyperactivity disorders and different EDCs including BCPs and pesticides such as chlorpyrifos has been done (de Cock et al., 2012).

The function of central nervous system (CNS) can be affected by EDCs and these effects can be induced by different mechanisms. The most important is the effects of EDCs on different endocrine axis important for CNS functions and development. Evidence that prenatal estrogen exposure is important in neuronal correct development emerged from reports of psychosis in patients prenatally exposed to the synthetic estrogen DES (Katz et al., 1987; Brown, 2009; Inadera, 2015; Negri-Cesi, 2015). Interestingly, several researches indicate that BPA is an estrogenic EDC that alters or interferes with normal endocrine development in various vertebrate and invertebrate species (vom Saal et al., 2007) suggesting a role of BPA in CNS disease. For instance, prenatal exposure to low dose of BPA disturbed neocortical histogenesis in mice (Nakamura et al., 2006, 2007).

As exposed above, BPA is a well-known xenoestrogen (Kuiper et al., 1998; Delfosse et al., 2014; Inadera, 2015). BPA has complex action in the CNS but primarily BPA was exhibited to bind both estrogen receptors α and β (ERα and ERβ) and has also been shown to act as an anti-androgen (Kuiper et al., 1998; Wolstenholme et al., 2011). Interestingly, it has been described endocrine and neuroendocrine abnormalities in schizophrenia (Marx and Lieberman, 1998; Stevens, 2002). In fact, estrogen has been associated with a neuroprotective effect but lower plasma levels of estrogens induced schizophrenia-like syndrome in males and females (Huber et al., 2001; Kaneda and Ohmori, 2005; Segal et al., 2007). Furthermore, neuronal disorders have also been associated with an impairment of HPA axis. For instance, the increase of glucocorticoid concentrations induced hippocampal nerve damage and schizophrenia (Cotter and Pariante, 2002). In rat, corticosterone exposures also lead to degeneration of the prefrontal cortex causing impairments in executive functions such as behavioral flexibility and working memory (Cerqueira et al., 2005). It has been established in baboons that HPA is potentially affected by estrogen (Pepe and Albrecht, 1998; Albrecht et al., 2005). In addition, it has been recently shown that perinatal exposure to low-dose of BPA caused HPA axis dysfunctions (Panagiotidou et al., 2014; Chen et al., 2015; Zhou et al., 2015). Particularly, the administration of low doses of BPA (2 μg/kg.day) to female breeders from gestation day 10 to lactation day 7 induced obvious anxiety/depression-like behaviors in the offspring (Chen et al., 2015). Notably, significant increase in serum corticosterone and adrenocorticotropin, and corticotropin-releasing hormone mRNA were detected in BPA-exposed rats before or after the mild stressor (Chen et al., 2015). Altogether these different observations strongly suggest that BPA and other EDCs could be associated to schizophrenia pathogenesis (Brown, 2009).

### Metabolic disorders

In addition to the reproductive and neuronal developmental effects, there is also evidence that metabolic disorders may be linked to EDCs (Casals-Casas et al., 2008; Newbold et al., 2008). Obesity, diabetes and metabolic syndrome are due to disruption of the energy storage balance endocrine system and thus are potentially sensitive to EDCs. This hypothesis is supported by different epidemiological and animal studies that have shown that a variety of EDCs can influence adipogenesis and obesity (Baillie-Hamilton, 2002; Casals-Casas et al., 2008; Elobeid and Allison, 2008; Newbold et al., 2008; Chen et al., 2009). For instance, the administration of DES to neonatal mice induced overweight associated with an increase of abdominal body fats and inflammatory biomarkers (Newbold et al., 2007). In rats, perinatal exposure to low doses of BPA increased adipogenesis and body weight in adult females (Somm et al., 2009). EDCs are also involved in glucose homeostasis defects. In accordance with this fact, epidemiological studies report that exposure to EDCs may affect the risk of type 2 diabetes (Remillard and Bunce, 2002; Huang et al., 2015; Song et al., 2016). Very low doses of BPA induced hyperinsulenemia and type 2 diabetes (Alonso-Magdalena et al., 2010). In the same way, low doses of BPA and dioxins altered α-cell function and glucagon release which lead to glucose homeostasis defect (Alonso-Magdalena et al., 2005). Interestingly, it has been established that EDCs such as BPA or dioxins are accumulated by adipose tissue and that they are released slowly and have induced glucose homeostasis impairment (Alonso-Magdalena et al., 2011). When administrated to mother mice, BPA induces metabolic disorders in adult male offspring such as an age-related change in food intake, an increase in body weight and liver weight, abdominal adipocyte mass, number and volume, and in serum leptin and insulin, but a decrease in serum adiponectin and in glucose tolerance (Angle et al., 2013). Furthermore, mother mice treated with BPA during gestation, at environmentally relevant doses, exhibit profound glucose intolerance and altered insulin sensitivity as well as increased body weight (Alonso-Magdalena et al., 2015).

## Micro-RNAs and EDCs

EDCs often act via more than one mechanism. The target cells of the hormones bear receptors specific to a given hormone and will be activated by either a lipid-soluble (permeable to plasma membrane) or water-soluble hormone (binds cell-surface receptor; Casals-Casas and Desvergne, 2011; Wolstenholme et al., 2011; Maqbool et al., 2016). Lipid-soluble hormones (steroid hormones and hormones of the thyroid gland) diffuse through the plasma membrane to enter the target cell and bind to a nuclear receptor (NR) protein that will in turn activates expression of specific genes that influence specific physiological cell activities. Water-soluble hormones (such as insulin) bind to a receptor protein on the plasma membrane of the cell which leads to specific cellular transduction pathways (Casals-Casas and Desvergne, 2011; Maqbool et al., 2016; Wolstenholme et al., 2011). Because many EDCs are small lipophilic compounds, they can directly interact with a given NR, which presumably perturbs or modulates downstream gene expression.

In parallel with these classical pathways, it appears that EDCs not only involve genetics but also epigenetic mechanisms. Epigenetics is broadly defined as those heritable changes in the genome not dependent upon changes in genetic sequences (e.g., DNA methylation or histone modification). These epigenetic processes control tissue development by controlling gene expression. Thus, a major route by which hormones act during development is by changing the epigenome. These different epigenetic mechanisms also include miRNAs which are short non-coding RNA molecules that post-transcriptionally repress the expression of genes by binding to 3′-untranslated regions (3′UTR) of the target mRNAs. Recently, it appears that miRNAs can be involved in the action of EDCs (Cameron et al., 2016; Klinge, 2015). This part of the review focuses on the regulation of miRNAs by the EDCs which appear as a new molecular mechanism involved in endocrine disruption.

### Biogenesis and action of miRNAs

The miRNAs are short non-coding RNA with a size of 21–26 nucleotides that suppress target gene expression through the inhibition of gene translation and the increase of the degradation of target mRNAs (Bartel, 2004). These small regulatory molecules are involved in a large range of biological processes such as development, cell proliferation, apoptosis, synaptic plasticity, and energy metabolism (Bartel, 2004). The gene regulation and processing as well as the mode of action of miRNAs are conserved over the evolution of a species (Stricklin et al., 2005; Landgraf et al., 2007; Ruby et al., 2007b). In recent decades, research on miRNAs has deepened our understanding of their mechanisms of action and their biological functions. These regulatory RNAs are predicted to modulate the expression of ~30% of protein-coding genes (Lewis et al., 2005). The miRNA can affect translation and mRNA stability by means of RNA-RNA interactions. A number of algorithms allow the identification of the potentially targeted mRNA by miRNA and conversely miRNA modulator of mRNA. Although the regulation of genes by miRNAs is an active area of research, few targets of miRNAs have been experimentally validated in a physiological context.

**Figure 1:**
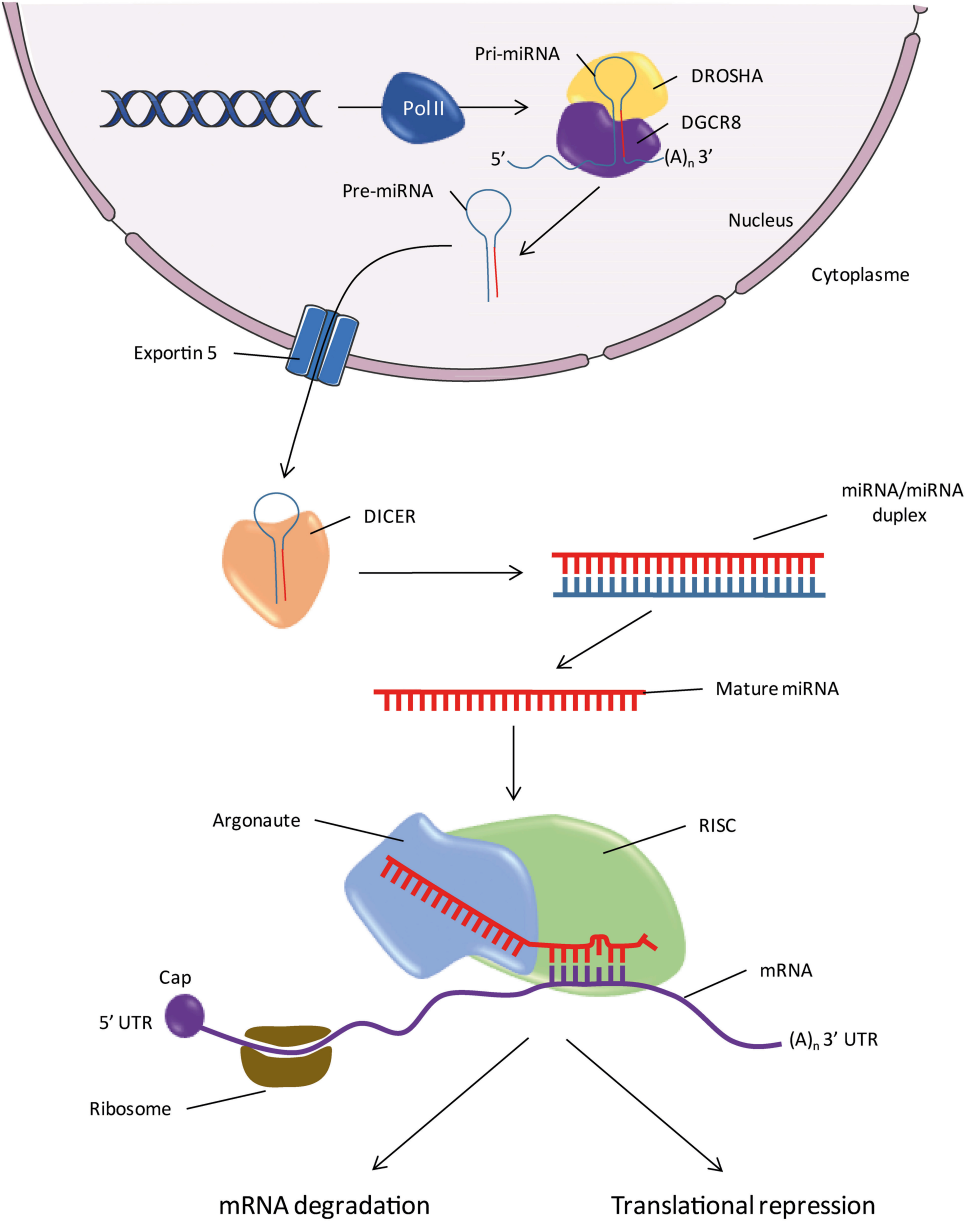
**Biogenesis of miRNAs**. Pol II, RNA Polymerase II; pri-miR, primary miRNA; pre-miR, precursor miRNA; RISC, RNA-Induced Silencing Complex; 5′ or 3′UTR, 5′ or 3′ untranslated region; DGCR8, DiGeorge Syndrome Critical Region 8; (A)_n_, Polyadenylation.

**Figure 2:**
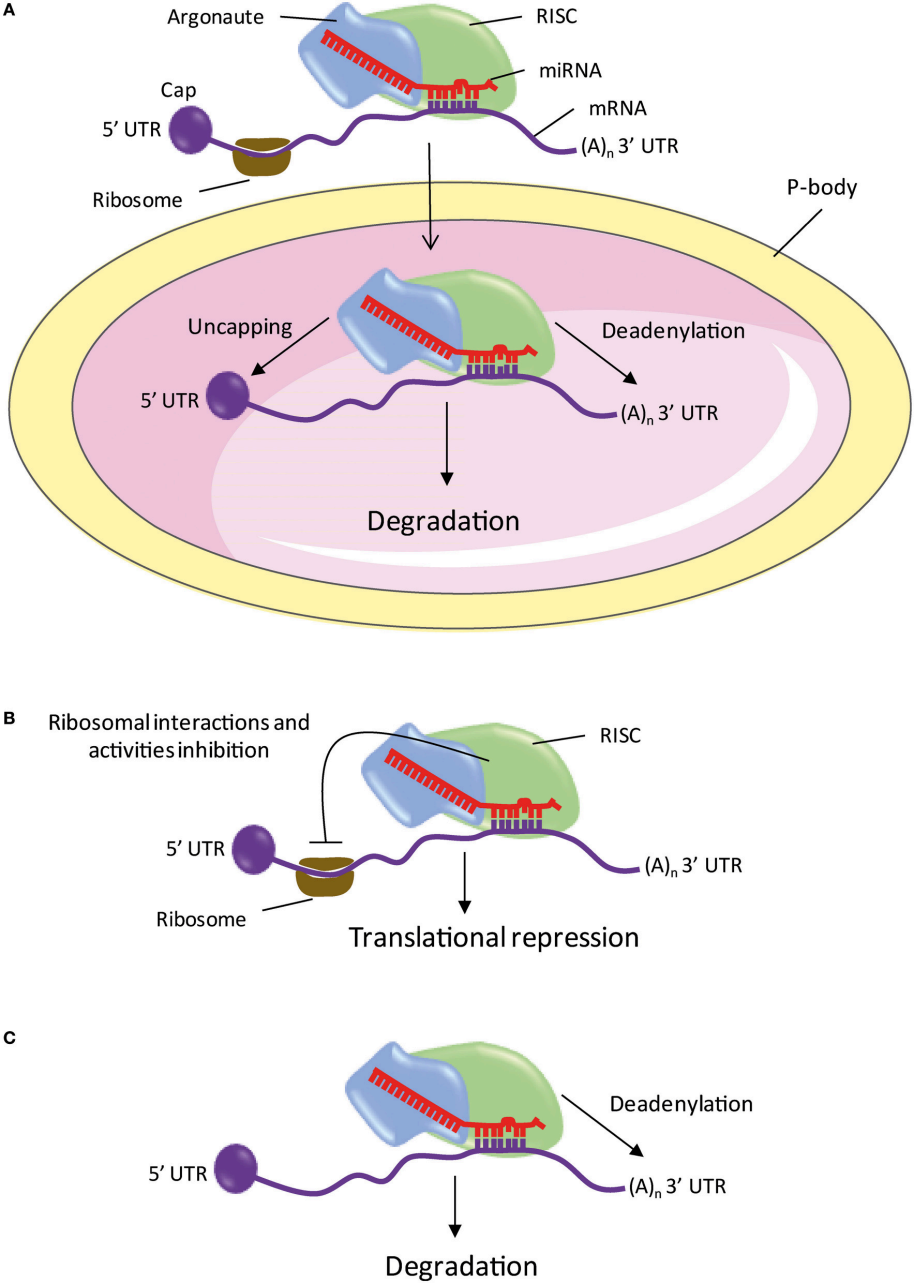
**The different mechanisms of mRNA repression or degradation by miRNA**. **(A)** The processing bodies. **(B)** Action on the initiation of translation and repression in post-initiation steps. **(C)** Deadenylation. miRNA, microRNA; P-bodies, processing bodies; RISC, *RNA-Induced Silencing Complex;* 5′ or 3′UTR, 5′ or 3′ untranslated region; (A)_n_, Polyadenylation.

In parallel with the discovery of new miRNA, the identification of the components of the miRNA maturation and processing machinery is an active area of research (Figure 1). The miRNA genes are located throughout the genome, within introns of protein-coding genes and rarely in exons (Rodriguez et al., 2004). Despite the small number of cases studied, it seems that the promoters of miRNAs have the same characteristics as those genes encoding proteins. The genes encode primary RNA (pri-miRNAs) conformation stem-loop, with one or two sequences which produce mature miRNAs (Hutvágner et al., 2001; Lagos-Quintana et al., 2001; Lau et al., 2001). The transcription machinery involves a RNA polymerase II (Lee et al., 2004; Bortolin-Cavaillé et al., 2009). The pri-miRNA is cleaved and polyadenylated at 3′ and 5′ capped in the same manner as the mRNAs (Figure 1; Cai et al., 2004). The steps of the pri-miRNA maturation require two endonucleases before they become functional miRNAs (Lee et al., 2003). The first step involves an RNA binding protein, the DiGeorge Syndrome Critical Region 8 (DGCR8) also called Partner of Drosha (PASHA) associated with Drosha (Denli et al., 2004; Gregory et al., 2004; Han et al., 2004). Drosha cleaves sequences on either side of the stem-loop of the pri-miRNA and gives the precursor miRNA (pre-miRNA). Pre-miRNA is exported from the nucleus to the cytoplasm by a karyopherin known as Exportin 5 (Yi et al., 2003; Bohnsack et al., 2004; Lund et al., 2004). In the second step, endonuclease DICER cleaves the pre-miRNA loop region in the cytoplasm, thereby releasing a double-stranded RNA of about 20 nucleotide pairs containing the mature miRNA (Bernstein et al., 2001; Grishok et al., 2001; Hutvágner et al., 2001; Ketting et al., 2001). Like Drosha, DICER is associated with an RNA binding-protein, the human immunodeficiency virus Transactivating Response RNA-Binding Protein (TRBP; Chendrimada et al., 2005; Gregory et al., 2005; Haase et al., 2005). One of the two strands is recognized by a protein of the family of the Argonautes (AGO), most commonly AGO2, which in turn recruits other elements of the RNA-induced silencing complex (RISC; Sontheimer, 2005). The other strand called “star strand” is degraded. An asterisk is associated with the name of the miRNA that is not incorporated into the RISC complex (e.g., miR-488*). However, for some miRNAs, both strands may be incorporated into the RISC complex. In this case, the end of the strand 5′ of the stem-loop is called “5p” and that of strand 3′ is called “3p” (e.g., miR-384-5p and miR-384-3p). In fact, new data indicates that a small fraction of the star strand is incorporated into the RISC complex for most miRNA families (Yang et al., 2011). For these reasons, the nomenclature scheme “–5p/–3p” is increasingly used instead of the terminology “mature/star.” The RISC complex/mature miRNA (miRISC) recognizes the target mRNA and induces degradation and/or inactivation of the latter (Figure 2).

However, various studies reveal that some families of miRNAs undergo non-canonical pathway maturation. Importantly, some studies described miRNAs called mirtrons which are located in the short sequence of introns. The mirtrons undergo a first processing step, independent of Drosha, by the splicing machinery to give miRNA with a lariat structure. The introns are then processed by the lariat-debranching enzyme to give the pre-miRNAs which carry-on its maturation by the canonical pathway (Okamura et al., 2007; Ruby et al., 2007a). It has also been reported in one case (miR-451) that the cleavage step by DICER is substituted with AGO2 (Cheloufi et al., 2010; Cifuentes et al., 2010).

### The miRNA-mRNA interactions

The action of miRNAs depends on their specific interaction with their targets. In plants, miRNAs bind to their targets with perfect complementarity of bases, which induces a rapid cleavage of the transcript by the ribonuclease activity of AGO (Baumberger and Baulcombe, 2005). In metazoans, the majority of miRNAs partially bind to their targets primarily through a region of so-called seed sequence, located at positions 2–7 from the miRNA 5′-end (Doench and Sharp, 2004; Brennecke et al., 2005). This region binds perfectly on the 3′UTR via complementary base interactions. These interactions induce inhibition of the expression of the target mRNA through a blocking of the translation or a degradation of the transcript. The different mechanisms of mRNA repression or degradation by miRNA are briefly described below.

Processing bodies (P-bodies) are cytoplasmic foci containing mRNA degradation enzymes and trinucleotide repeat-containing gene 6A protein (TNRC6A or GW182 for Drosophila). These are involved in the catabolism and/or storage of untranslated mRNA (Figure 2A; Eystathioy et al., 2002, 2003; Ingelfinger et al., 2002; van Dijk et al., 2002; Sheth and Parker, 2003). The GW182 proteins are also found in the miRISC complex where they play a key role in the repression induced by miRNAs (Jakymiw et al., 2005; Liu et al., 2005; Eulalio et al., 2008). In addition, the AGO and GW182 proteins, miRNAs and targeted mRNAs are found in the P-bodies (Ding et al., 2005; Liu et al., 2005; Pillai et al., 2005; Sen and Blau, 2005). These studies suggest that targeted mRNAs are repressed or degraded in the P-bodies.

The mechanism by which miRISC inhibits translation is controversial. Several studies indicate a blocking of the initiation of translation, while other studies suggest a repression in post-initiation steps (Figure 2B). Indeed, it has been shown that the miRNA targeted mRNAs are associated with fewer ribosomes during elongation than in mRNAs controls (Humphreys et al., 2005; Pillai et al., 2005; Bhattacharyya et al., 2006; Huang et al., 2007; Ding and Grosshans, 2009). The initiation is stopped by the blocking by miRISC of the interaction of the translation ribosomal subunit 60S with mRNA (Chendrimada et al., 2007; Wang et al., 2008). In addition, GW182 recognizes the 5′ cap of the mRNA and prevents the initiation of translation (Eulalio et al., 2008). In the other studies, two mechanisms inducing translation repression after initiation have been described. It has been shown that miRISC promotes the release of ribosomes during elongation, thus blocking translation (Petersen et al., 2006). Another study suggests that the elongation process is maintained without peptide production when mRNA is targeted by a miRNA (Nottrott et al., 2006). The authors suggest that the complex-related proteases miRISC could degrade the native peptides.

Studies showed that repression of many miRNA targets is associated with a deadenylation and degradation (Figure 2C; Lim et al., 2005; Giraldez et al., 2006; Wu et al., 2006; Wakiyama et al., 2007; Eulalio et al., 2009). Comparative analysis of large scale proteomic and transcriptomic changes, following overexpression or inhibition of a miRNA in mammalian cells show that the vast majority of targets repressed by a miRNA have decreased their level of mRNA reflecting a lower presence of protein (Baek et al., 2008; Selbach et al., 2008; Hendrickson et al., 2009; Guo et al., 2010). These studies show that repression induced by miRNAs predominantly results in mRNA degradation.

### Modulation of miRNA expression by hormones

Numerous studies clearly indicated that different hormones modulate miRNA expression in different organs (Hu et al., 2013; Cameron et al., 2016; Derghal et al., 2015; Klinge, 2015). For instance, the treatment with thyroid hormones of hepatocytes cells AML 12 over-expressing miR-206 resulted in decreased miR-206 expression, and a significant increase in two predicted target genes (i.e., Mup1 and Gpd2; Dong et al., 2010).

It has also been shown that estradiol actively controls miRNA production in various tissues such as mammary and ovarian cells (Gupta et al., 2012). More precisely, estrogens modulate miRNA transcription by inactivating RNA polymerase II and precursor miRNA biogenesis by blocking Drosha-mediated processing (Gupta et al., 2012). It also been shown that estrogen regulates miRNA expression in brain and particularly in the hippocampus, the amygdala and paraventricular nucleus (Rao et al., 2013). Recently, it has been established that miR-27a/b and miR-494 regulate tissue factor pathway inhibitor α (TFPIα) expression suggesting a possible role of these miRNAs in the estrogen mediated downregulation of TFPIα involved in breast cancer (Ali et al., 2016).

Several studies indicate that gonadotropins as estrogen can affect miRNA expression (Cohen et al., 2016). In accordance with this, it has been observed variability in miRNA expression profiles in estrogen receptor-positive and -negative breast cancer phenotypes (Iorio et al., 2005; Mattie et al., 2006). As described recently miR-136-3p expression levels were increased after the administration of human chorionic gonadotropin to ovarian cells (Kitahara et al., 2013). Direct action of estrogen on miRNAs expression has been demonstrated in different studies. For instance, an aberrant miRNA expression has been characterized in estrogen-induced rat breast carcinogenesis (Kovalchuk et al., 2007). Using the microarray approach, it has been shown that estrogen can modulate the profile of miRNAs expression in zebrafish model and in human MCF-7 and ZR-75 breast cancer cells (Cohen et al., 2008; Bhat-Nakshatri et al., 2009; Maillot et al., 2009; Ferraro et al., 2012).

Altogether, these different observations suggest that the link between hormones, miRNAs and mRNA targets will lead to an improved understanding of how EDCs affect the different endocrine axis.

### Modulation of miRNA expression by EDCs

A few recent studies report the effect of several EDCs on the expression of miRNAs in fish, animals, or cell lines (Collotta et al., 2013; Vrijens et al., 2015). These disturbances of miRNAs expression profile by EDCs are associated with diseases of the CNS and reproductive axis as well as metabolic disorders (Vrijens et al., 2015).

In humans, it has been shown that several EDCs as DTT or BPA decreased the expression of miR-21 which has a key role in cancer especially in breast cancer development (Tilghman et al., 2012; Sicard et al., 2013). In addition, decreased expression of let-7f is also associated with breast cancer (Sakurai et al., 2012). In the work led by Tilghman et al., DTT (10 μM) or BPA (10 μM) activate ERα in MCF-7 breast cancer cells which down-regulated the expression of miR-21, let-7a-f, miR-15b, and miR-28b and increased the expression miR-638, miR-663, and miR-1915 (Tilghman et al., 2012). In addition, it has been exhibited an important role of miR-19 in BPA-mediated MCF-7 cell proliferation (Li et al., 2014). The xenoestrogens DES also showed a decrease of miR-34b expression in MCF-7 cells (Lee et al., 2011). In rats, the neonatal exposure to the estrogenic analog (i.e., estradiol benzoate) increased the expression of miR-29 in testicular tissue (Meunier et al., 2012). Increased miR-29 expression resulted in a decrease in DNA methyltransferases (DNMT1, 3a and 3b) and antiapoptotic myeloid cell leukemia sequence 1 (Mcl-1) protein levels. Together, the increased miR-29 combined with a subsequent reduction of DNMT and Mcl-1 protein levels may represent a basis of explanation for the adult expression of the germ cell apoptosis phenotype. Interestingly, BPA given to rats at moderate doses is associated with erectile dysfunction, cavernosal lipofibrosis and alterations of global gene transcription including a set of miRNAs expressed in the penile shaft (Kovanecz et al., 2014). In female, prenatal BPA treatment in sheep results in hypergonadotropism and ovarian cycle disruptions (Veiga-Lopez et al., 2013). Interestingly, in this study it has been shown that fetal ovarian miRNAs expression was altered by prenatal BPA with 45 down-regulated (>1.5-fold) at day 65 and 11 down-regulated at day 90 of gestation (Veiga-Lopez et al., 2013). In chicks, several miRNAs (miR-1623, miR-1552-3p, miR-1573, miR-124a, and miR-1764) were down-regulated in the DES-treated chick oviduct compared with control oviduct (Lim and Song, 2015). Interestingly, these miRNAs regulate the expression of vitelline membrane outer layer protein 1, a basic protein present in the outer layer of the vitelline membrane of eggs, plays essential roles in separating the yolk from the egg white (Lim and Song, 2015). There is a growing concern about the potential health effects of exposure to various EDCs during pregnancy and infancy. The placenta is expected to be an effective barrier protecting the developing embryo against some EDCs circulating in maternal blood. However, it has been shown recently that miR-146a was significant overexpressed and correlated significantly with BPA accumulation in the placenta from pregnant women living in a polluted area and undergoing therapeutic abortion due to fetal malformations (De Felice et al., 2015). This observation has been also established in HTR-8 and 3A human placental cells (Avissar-Whiting et al., 2010). These different studies highlight the fact that the EDCs induce miRNA-expression alterations in the reproductiveaxis.

In the context of CNS disease, Jiang et al. established by *in silico* approach that miR-146a is involved in Alzheimer's disease (Jiang et al., 2013). Interestingly, BPA exposure of human placental cell lines has been shown to alter miRNA expression levels, and specifically, miR-146a was strongly induced by BPA treatment (Avissar-Whiting et al., 2010). Then, miR-146a could be used as a biomarker for Alzheimer's disease after EDCs exposure.

Recently, it has been established that the expression of hepatic miRNA (miR-22b, miR-140, miR-210a, mir-301, miR-457b, and let-7d) is increased in fluoxetine (the active ingredient in Prozac®) exposed female zebrafish (Craig et al., 2014). Interestingly, the miRNAs that were up-regulated were predicted to be responsible for down-regulating pathways such as insulin signaling, cholesterol synthesis, and triglyceride synthesis (Craig et al., 2014). Recently, it was shown that miR-21, 221, 222, and 429 expression levels decreased in the liver of DDT-treated female Wistar rats, whereas increases were observed in cytochrome 1A1 and 2B1 mRNA (Chanyshev et al., 2014; Gulyaeva et al., 2016). By an original approach using DNA-Au bio bar code (DNA-Au) and G-quadruplex-based DNA enzyme, Meng et al. demonstrated that miR-21 expression is increased in BPA-treated human hepatocarcinoma BEL-7402 cells (Meng et al., 2013). In primary mouse hepatocyte, TCCD modulated the expression of miR-503-5p that targeting cyclin D2 which was involved in the discriminative process of p53 signaling and metabolism (Rieswijk et al., 2015). In addition, it also been shown that TCDD regulates the expression of miR-101a and miR-122 and that cyclooxygenase-2, a target gene of miR-101a, plays a significant role in liver damage in mice exposed to TCDD (Yoshioka et al., 2011). Altogether, these observations suggest that the EDCs can induce metabolic disorders through the disturbance of specific miRNAs in the liver.

Altogether, these different studies indicated that miRNAs profile changed in tissue exposed to different EDCs. Potentially, miRNAs can be considered as new biomarkers for EDCs exposure (Vrijens et al., 2015).

## Conclusion

Despite the high number of studies generated in the past few years on the mechanism of how EDCs act on the different endocrine axis, much still needs to be learnt. To date, very few ecotoxicology studies have considered miRNA in the context of endocrine disruption. In this review, we have seen that exposure to EDCs may lead to modification of miRNAs expression associated with endocrine disruption. However, many questions remain open, for instance (i) what is the impact on the miRNAs expression in different tissues which have suffered chronic low level EDCs exposure, (ii) what are the effects of the exposure either to a single EDC or to a complex mixture of different chemicals. Further, studies are warranted to evaluate if miRNAs may act as a causal link between EDCs exposure and their effect on health or if they can be used as a diagnostic or prognostic tools.

## Author contributions

LM and AD wrote the manuscript. MD and JT helped with manuscript preparation.

### Conflict of interest statement

The authors declare that the research was conducted in the absence of any commercial or financial relationships that could be construed as a potential conflict of interest.
